# Influence of motivation on rehabilitation outcomes after subacute stroke in convalescent rehabilitation wards

**DOI:** 10.3389/fneur.2023.1185813

**Published:** 2023-07-14

**Authors:** Taiki Yoshida, Yohei Otaka, Shin Kitamura, Kazuki Ushizawa, Masashi Kumagai, Jun Yaeda, Rieko Osu

**Affiliations:** ^1^Tokyo Bay Rehabilitation Hospital, Department of Rehabilitation Medicine, Chiba, Japan; ^2^Faculty of Rehabilitation, School of Health Sciences, Fujita Health University, Aichi, Japan; ^3^Department of Rehabilitation Medicine I, Fujita Health University School of Medicine, Aichi, Japan; ^4^Graduate School of Comprehensive Human Science, University of Tsukuba, Tokyo, Japan; ^5^Faculty of Human Sciences, Waseda University, Saitama, Japan

**Keywords:** motivation, functional independence measure, stroke, hospitalization, rehabilitation

## Abstract

**Background:**

The motivation for rehabilitation is important in encouraging stroke patients to participate in rehabilitation; however, its relationship with outcomes is not well known. In addition, changes in patient motivation during hospitalization have not been examined.

**Aim:**

To examine the relationship between motivation and rehabilitation outcomes for subacute stroke patients and to investigate the changes in motivation.

**Design:**

Prospective cohort study.

**Setting:**

Subacute rehabilitation hospital.

**Population:**

The study enrolled a consecutive sample of patients (*n* = 201) with stroke admitted to a subacute rehabilitation ward from October 2017 to March 2019.

**Methods:**

The functional independence measure and motivation in stroke patients for rehabilitation scale was evaluated at admission; at one, two, and three months after admission; and at discharge. The effectiveness and efficiency of the functional independence measure were calculated as rehabilitation outcomes. The effect of motivation on outcomes and the change in motivation in stroke patients for rehabilitation scale scores over time were analyzed using a linear mixed model.

**Results:**

The median (interquartile range) converted motivation in stroke patients for rehabilitation scale scores (converted to a range of 0–100) at admission; one, two, and three months after admission; and discharge was 86 (76–95), 83 (77–94), 81 (74–95), 81 (71–93), and 84 (75–95), respectively. The median (interquartile range) of effectiveness and efficiency of the functional independence measure from admission to discharge was 0.82 (0.68–0.91) and 0.41 (0.30–0.59), respectively. Motivation in stroke patients for rehabilitation scale scores were not significantly associated with the effectiveness and efficiency of the functional independence measure (*p* > 0.05). Motivation in stroke patients for rehabilitation scale scores were significantly lower at two (*β* = −3.1, 95% confidence interval [−5.3, −0.9], *p* = 0.005) and three (*β* = −4.4, 95% confidence interval [−7.3, −1.6], *p* = 0.002) months after admission than at admission.

**Conclusion:**

Motivation might not directly affect rehabilitation outcomes assessed by the functional independence measure. Furthermore, many participants remained highly motivated, although their motivation decreased at one or three months after admission.

**Clinical rehabilitation impact:**

Assumptions that rehabilitation is ineffective because of low motivation may not be correct. To examine the influence on outcomes, both motivation and daily activities should be considered.

## 1. Introduction

Motivation is described as an essential factor in rehabilitation outcomes and is a concept frequently used by rehabilitation professionals (1). It plays an important role in enhancing the continuity of rehabilitation programs (1, 2). Moreover, motivation is a factor that increases opportunities for self-training and improves the amount of physical activity performed during hospitalization (3). Stroke patients’ motivation for rehabilitation is influenced by interactions with rehabilitation professionals; therefore, it is possible to modify their motivation based on how the professionals interact with them (3). Furthermore, this change in motivation could be greater among inpatients, as they have frequent opportunities to communicate with rehabilitation professionals. Since physical activity promoted by motivational status is associated with improved outcomes (4, 5), motivation for rehabilitation could influence the rehabilitation outcomes of the patients. Motivation could have more impact during the subacute phase of stroke when the ability to improve physical function is higher. However, there is currently no research on the relationship between motivation and improved rehabilitation outcomes during the subacute phase of stroke.

Several previous studies have reported the relationships between motivation and outcomes of stroke survivors other than those during the subacute phase (6–8). Studies focusing on the late subacute to chronic phase have shown, using qualitative methods, that motivation plays an important role in sustaining physical activity (6–8). However, no report has investigated any specific correlation between participant motivation and performance. Furthermore, the relationship between motivation and rehabilitation outcomes was assessed using the intrinsic motivation items of the Multidimensional Health Locus of Control and the independent activities of daily living (9). However, this evaluation scale is not specific to motivation for rehabilitation. Another previous study reported that patients considered by medical staff to have higher motivation showed improvements in physical function through physical therapy at three months and six months after stroke onset (10). However, studies have shown that the results of observational evaluations by others, such as medical staff, may lead to mislabeling of participants (1, 3).

Psychological conditions related to lack of motivation include depression and apathy. The symptoms of apathy include a loss of motivation, while depression symptoms involve a lack of interest in activities related to motivation (11–14). Previous studies have objectively evaluated depression and apathy using assessment scales and reported negative impacts on rehabilitation outcomes (15, 16). Hence, there is a possibility that a decrease in motivation could have a negative effect on rehabilitation results. However, as previously reported, there are individuals in states such as depression and apathy who exhibit high motivation for rehabilitation, as well as individuals who lack motivation for rehabilitation despite not experiencing depression or apathy (17). Therefore, it suggests that the motivation for rehabilitation contains elements that cannot be measured by scales assessing depression or apathy. There have been no reports investigating the correlation between rehabilitation-specific motivation and rehabilitation outcomes, rather than focusing on apathy and depression.

Previous studies reporting associations between motivation and rehabilitation have focused on the acute (9), chronic (18, 19), and late subacute to chronic phases after stroke onset (6–8, 10); however, no study has focused on the early subacute phase of stroke, which can be defined as seven days to three months after stroke onset (20), when physical function and abilities are markedly improved. In Japan, convalescent rehabilitation wards called Kaifukuki Rehabilitation Wards (KRWs) provide intensive rehabilitation for stroke patients during the subacute phase for two to three months (21). Daily intensive training (up to 3 h) is conducted in the rehabilitation wards during the long hospitalization period. The motivation of patients admitted to rehabilitation hospitals is influenced by multiple factors (3). In other words, patients’ motivation is situation-dependent and may readily become unstable. However, there have been no reports measuring the state of patients’ motivation toward rehabilitation over time. For the medical staff to effectively intervene in the motivation of patients undergoing rehabilitation, they must understand how patients’ motivation changes during hospitalization.

One of the potential reasons the relationship between motivation and rehabilitation outcomes has not yet been well-investigated is the lack of evaluation scales focusing on motivation for rehabilitation. We developed an evaluation scale for motivation toward rehabilitation referred to as the Motivation in stroke patients for rehabilitation scale (MORE scale) during our previous study (17). This scale quantitatively assesses motivation for rehabilitation based on the patients’ answers and can comprehensively assess not only the motivation for training but also the patients’ relationship with rehabilitation professionals and attitudes regarding discharge.

In this study, we investigated our hypothesis that during the subacute phase, stroke patients’ motivation toward rehabilitation affects the outcomes of the convalescent rehabilitation provided in the hospital. We also examined changes in motivation over time using the MORE scale to understand the appropriate intervention timing.

## 2. Materials and methods

### 2.1. Study design

This study had a prospective cohort design. The study protocol was approved by the Ethics Committee of the Tokyo Bay Rehabilitation Hospital (approval number: 144; date of approval: October 13, 2016) and the Ethics Committee of Waseda University (approval number: 2019–059; date of approval: June 23, 2019). All participants provided written and verbal informed consent prior to their participation in this study. This study was conducted in accordance with the principles set forth in the 1964 Helsinki Declaration, as revised in 2013, and was reported in accordance with the Strengthening the Reporting of Observational Studies in Epidemiology (STROBE) guidelines (22).

### 2.2. Participants

This study involved consecutive patients admitted to the subacute rehabilitation hospital from October 2017 to March 2019. The inclusion criteria were as follows: (1) first-ever stroke, (2) onset within two months of admission, and (3) no physical or cognitive problems that could interfere with the measurement of the MORE scale score. The exclusion criterion was having medical risks such as rapid deterioration of the patients’ condition. The participants in this study were the same as in the sample involved in our previous scale development study (21). In the previous study (21), the target sample size was set at 200, based on the number of participants necessary for structural validity verification with the assumption of missing values, according to the Consensus-based standards for the selection of health measurement instruments (COSMIN) (23, 24).

### 2.3. Study setting

This study was conducted in KRWs (21). The KRWs is a system for subacute rehabilitation in Japan and is covered by government medical insurance. Patients are admitted to an acute phase hospital after stroke onset and receive one–two months of treatment. After the acute phase treatment, patients are admitted to a rehabilitation hospital when the medical staff judges that they require additional rehabilitation treatment, except for mild cases that can be managed at home or the most severe cases. In KRWs, patients underwent daily one-on-one intensive rehabilitation sessions with therapists for approximately 2–3 h. The typical schedule was 1 h in the morning and 1–2 h in the afternoon. Patients also engaged in self-training sessions outside of the rehabilitation sessions if indicated. The training content with therapists and self-training was determined through discussions between the rehabilitation professionals and the patients.

### 2.4. Data collection

The following data were collected: patients’ gender, age, type of stroke, lesion side, days from onset to admission, length of hospital stay, Mini-Mental State Examination score (25), Stroke Impairment Assessment Set-motor function score (26), and the Functional Independence Measure (FIM) score (27). In addition, patients’ motivation for rehabilitation was assessed using the MORE scale (17). The FIM and MORE scales were evaluated at the following time periods: at admission, at one month after admission, at two months after admission, at three months after admission, and at discharge.

### 2.5. MORE scale

The MORE scale is a 17-item scale for evaluating stroke patients’ motivation toward rehabilitation. Each item is rated using a 7-point scale (1) strongly disagree, (2) disagree, (3) somewhat disagree, (4) neither agree nor disagree, (5) somewhat agree, (6) agree, and (7) strongly agree. The scores range from 17 to 119 points, with higher scores indicating higher motivation. The MORE scale was developed with reference to the factors influencing the motivation of stroke patients (17). In self-determination theory (28, 29), widely known as the motivation theory, motivation is broadly classified into intrinsic motivation and extrinsic motivation. According to this classification, rewards, including functional recovery and praise from medical staff and family members, can be categorized as extrinsic motivation, and patients’ enjoyment of the rehabilitation itself can be classified as intrinsic motivation. Since the motivation of hospitalized stroke patients for rehabilitation is mainly influenced by extrinsic factors (3), each item of the MORE scale focuses on the patients’ extrinsic factors. The validation and reliability of the MORE scale were established in stroke patients according to the COSMIN guidelines (23, 24).

### 2.6. Rehabilitation outcomes

The outcome measures of rehabilitation, FIM efficiency, and FIM effectiveness were calculated based on the FIM scores. The FIM gain is an index of the degree of improvement in FIM scores during hospitalization (30). FIM effectiveness reflected the degree of improvement in the FIM score within the range of possible improvement for hospitalized patients calculated as follows: FIM gain / (maximum score − admission score) (31, 32). FIM efficiency reflected the degree of improvement in FIM scores per day of hospitalization calculated as follows: FIM gain / length of hospitalization (days) (31, 32).

### 2.7. Analyses

Continuous values are shown as median (IQR: interquartile range), and categorical variables are shown as frequencies. The length of hospital stay varied among the participants. Consequently, it was necessary to consider missing data for the longitudinal data. Therefore, we utilized a linear mixed-effects model as the analytical method. To evaluate the relationship between the FIM effectiveness, efficiency, and MORE scale score, we performed a linear mixed-effects model with time points, MORE scale score, and their interaction (time points*MORE scale score) as fixed effects and with participants as a random effect. Random intercept and slope were used. A linear mixed-effects model was used to compare MORE scale scores over time, with time points as fixed effects and participants as random effects. Statistical analyses were performed using IBM SPSS Statistics version 27.0 (IBM Corp., Armonk, NY, United States) and R (version 3.6.1). Statistical significance was set at *p* < 0.05.

## 3. Results

**Figure 1:**
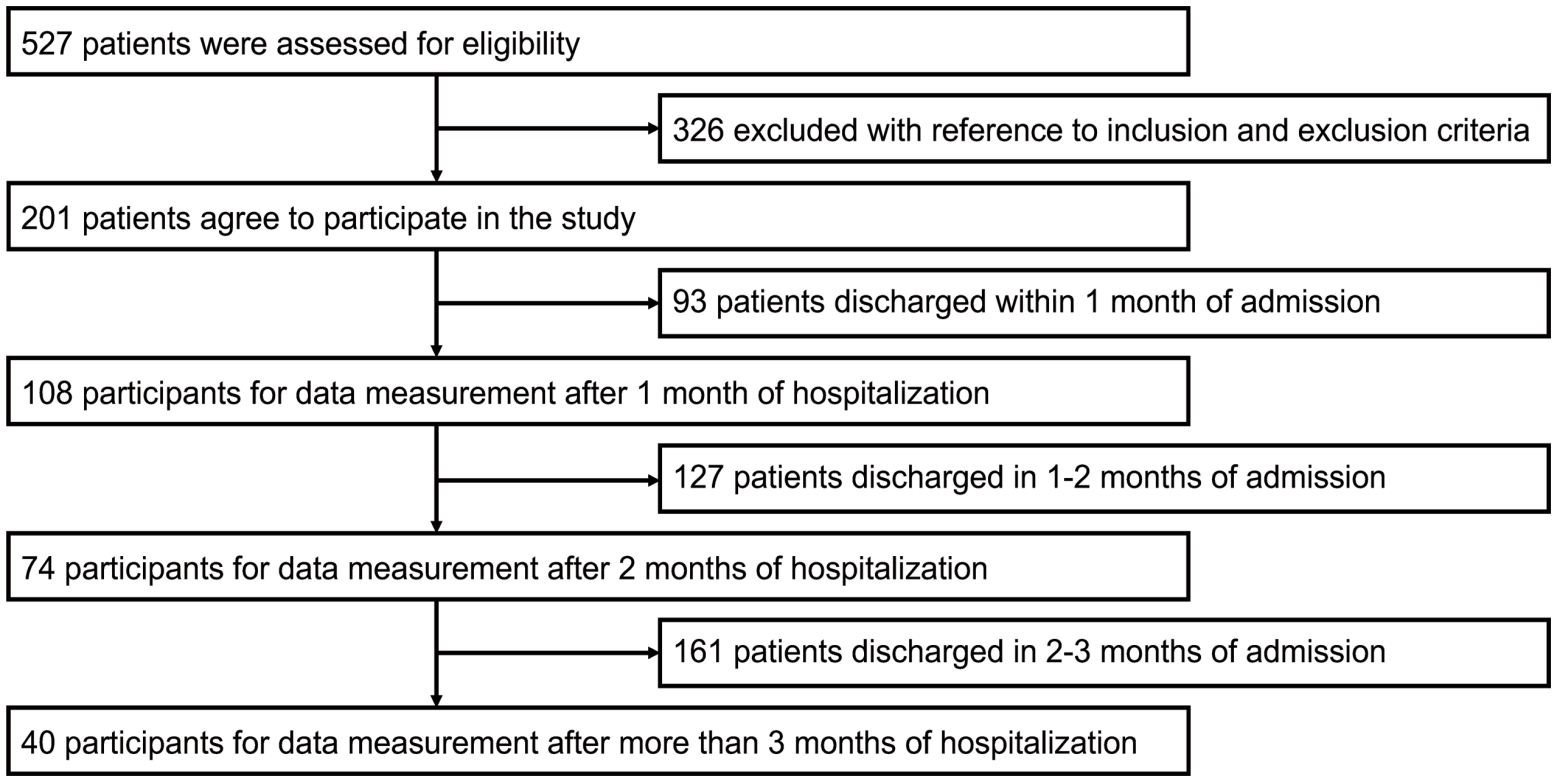
Participants inclusion flowchart.

From a consecutive series of 527 stroke patients hospitalized during our study period, 201 participants were selected for analysis (Figure 1). The participants were chosen based on the inclusion criteria, and consent was obtained. Of the 201 patients, 108 patients were evaluated after one month, 74 after two months, and 40 after three months over time. Table 1 presents the participants’ characteristics. The median (IQR) days after admission when each assessment was performed was nine (6–12) days for the admission evaluation, 41 (37–45) days for the one-month evaluation, 70 (67–75) days for the two-month evaluation, 100 (97–103) days for the three-month evaluation, and 63 (36–106) days for the discharge evaluation. The median (IQR) of total FIM gain, effectiveness, and efficiency from admission to discharge were 28 (21–39), 0.82 (0.68–0.91), and 0.41 (0.30–0.59), respectively. The median (IQR) of Mini-Mental State Examination score was 28 (25–29).

**Table 1:** Participants’ characteristics (*n* = 201).

Age, years, median (IQR)	67 (56–76)
Type of stroke, hemorrhage; infarction	127; 74
Lesion side, right; left; both	92; 105; 4
Days from stroke onset to admission, median (IQR)	28 (20–39)
Length of hospital stay, days, median (IQR)	64 (37–109)
Mini-Mental State Examination, median (IQR)	28 (25–29)
Stroke Impairment Assessment Set motor function, median (IQR)	
Knee-mouth test	4 (3–5)
Finger-function test	4 (2–5)
Hip-flexion test	4 (4–5)
Knee-extension test	4 (4–5)
Foot-pad test	4 (3–5)
FIM at admission, median (IQR)	
Total score	88 (74–100)
Motor score	61 (48–70)
Cognitive score	28 (25–32)
FIM at discharge, median (IQR)	
Total score	119 (112–124)
Motor score	87 (81–89)
Cognitive score	33 (30–35)
FIM gain from admission to discharge, median (IQR)	28 (21–39)
FIM effectiveness from admission to discharge, median (IQR)	0.82 (0.68–0.91)
FIM efficiency from admission to discharge, median (IQR)	0.41 (0.30–0.59)
Days from admission to evaluate MORE scale, median (IQR)	
At admission	9 (6–12)
1 month after	41 (37–45)
2 months after	70 (67–75)
3 months after	100 (97–103)
At discharge	63 (36–106)

IQR, interquartile range; FIM, functional independence measure; MORE scale, motivation in stroke patients for rehabilitation scale.

### 3.1. Influence of motivation on rehabilitation outcomes

The results of the association of the MORE scale, FIM effectiveness, and FIM efficiency are shown in Table 2. The results of the linear mixed-effects model with time points and MORE scale score as fixed effects and with participants as a random effect showed no significant association with FIM efficiency and effectiveness (*p* > 0.05). The main effect of time point was found (*p* < 0.05), with significantly lower FIM effectiveness and FIM efficiency from admission to two months, two to three months, and after three months compared to FIM effectiveness and FIM efficiency from admission to one month after. The interaction of time points and the MORE scale score was not found (*p* > 0.05).

**Table 2:** Association between converted MORE scale scores and rehabilitation outcomes assessed by FIM effectiveness and FIM efficiency using a linear mixed-effects model.

	FIM effectiveness			FIM efficiency			*β*	95%CI	*p-*value	*β*	95%CI	*p-*value
Converted MORE scale	−0.002	[−0.005 to 0.000]	0.060	−0.002	[−0.005 to 0.000]	0.122
Time points						
Admission to 2 months after [T2]	−0.446	[−0.819 to −0.072]	0.019	−0.760	[−1.085 to −0.434]	< 0.001
Admission to 3 months after [T3]	−0.655	[−1.064 to −0.247]	0.002	−0.890	[−1.268 to −0.513]	< 0.001
Admission to more than 3 months after [T4]	−0.576	[−1.017 to −0.137]	0.010	−0.726	[−1.164 to −0.287]	0.001
Converted MORE scale*T2	0.002	[−0.002 to 0.006]	0.308	0.002	[−0.001 to 0.005]	0.286
Converted MORE scale*T3	0.003	[−0.001 to 0.008]	0.194	0.002	[−0.001 to 0.007]	0.272
Converted MORE scale*T4	0.003	[−0.002 to 0.008]	0.263	0.001	[−0.003 to 0.006]	0.547

FIM, functional independence measure; MORE scale, motivation in stroke patients for rehabilitation scale; CI, confidence interval.

### 3.2. Changes in motivation for rehabilitation over time

**Figure 2:**
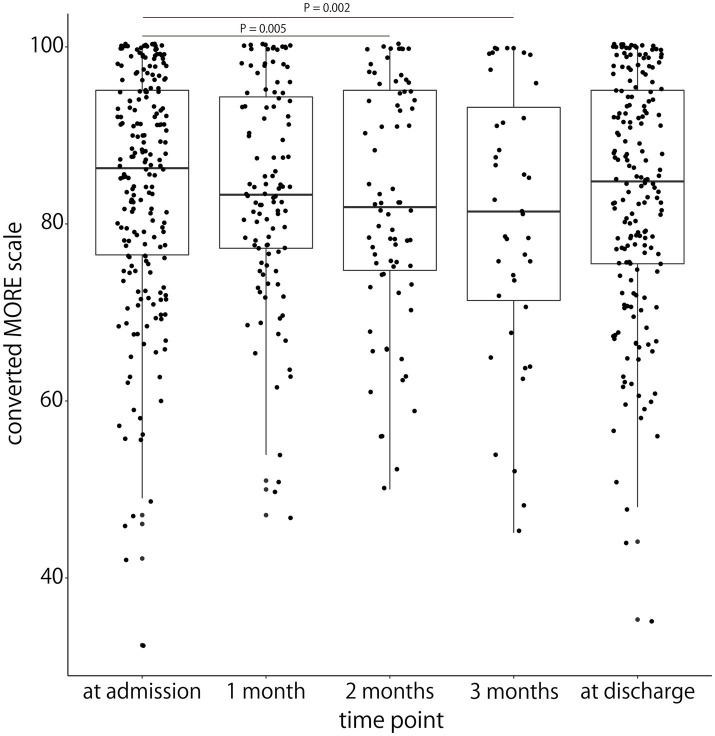
Comparison of the MORE scale scores at admission, at 1 month after admission, at 2 months after admission, at 3 months after admission, and at discharge. The *x*-axis represents each evaluation time point. The *y*-axis represents the MORE scale score converted to a score of 0–100. The horizontal line inside each box represents the median, the boxes extend to the lower and upper quartiles, and the whiskers extend to the extreme values. Each plot represents the participants’ individual data. Significant differences between admission and both 2 and 3 months after admission are shown.

The median (IQR) MORE scale scores in stroke patients admitted to the rehabilitation ward were 105 (95–114) at admission, 102 (95–113) at one month after admission, 100 (93–114) at two months after admission, 100 (89–112) at three months after admission, and 103 (94–114) at discharge. The lowest score of 17 on the MORE scale was converted to 0, and the highest score of 119 was converted to 100. This resulted in median (IQR) scores for each evaluation time point as follows: 86.3 (76.5–95.1) at admission, 83.3 (77.2–94.4) at one month after admission, 81.9 (74.8–95.1) at two months after admission, 81.4 (71.3–93.1) at three months after admission, and 84.8 (75.5–95.1) at discharge. The mixed linear-effects model showed significantly lower converted MORE scale scores after two months (*β* = −3.1, 95% confidence interval [CI]: −5.3 to −0.9, *p* = 0.005) and three months (*β* = −4.4, 95% CI: −7.3 to −1.6, *p* = 0.002) of hospitalization relative to those at the time of admission. Many participants remained highly motivated, although their motivation decreased two or three months after admission (Table 3 and Figure 2).

**Table 3:** Comparison using a linear mixed-effects model with the converted MORE scale at each time point.

Time points	Median (IQR)	*β*	95%CI	*p*-value
At admission	86.3 (76.5–95.1)	–	–	–
1 month	83.3 (77.2–94.4)	−1.05	[−2.95 to 0.85]	0.278
2 months	81.9 (74.8–95.1)	−3.15	[−5.34 to −0.95]	0.005
3 months	81.4 (71.3–93.1)	−4.48	[−7.31 to −1.65]	0.002
At discharge	84.8 (75.5–95.1)	−0.72	[−2.27 to 0.84]	0.365

IQR, interquartile range; CI, confidence interval; MORE scale, motivation in stroke patients for rehabilitation scale.

## 4. Discussion

This study examined the temporal changes in motivation for rehabilitation and the relationship between motivation and rehabilitation outcomes in stroke patients admitted to a convalescent rehabilitation ward. The results showed that the motivational status among relatively highly motivated participants was not associated with rehabilitation outcomes, as detected by the FIM effectiveness and efficiency. Regarding changes in motivation over time, the motivation for rehabilitation decreased after two and three months of hospitalization compared to motivation on admission; however, motivation remained relatively high throughout hospitalization.

Our initial hypothesis was that motivation could affect the rehabilitation outcomes, namely the FIM effectiveness and efficiency. However, the study could not confirm the association between stroke patients’ motivation and rehabilitation outcome measures during hospitalization. The finding that motivation was not associated with rehabilitation outcomes must be interpreted in light of two possibilities. The first is that many of the participants were highly motivated. A previous study reported a difference in improvement in physical function in the late subacute phase between highly motivated and less motivated patients (10). Thus, the possibility that low motivation for rehabilitation may have a negative impact on outcomes cannot be ruled out. In our study, most participants were relatively highly motivated. Therefore, the association between motivation and outcomes in participants with significantly low motivation could not be ascertained. The second possibility was that motivation might affect the indicators that were not detected in the FIM, such as physical fitness and quantity of daily activity. It may be possible to confirm the influence of motivation by analyzing indicators other than the FIM scores as rehabilitation outcomes, such as participants’ physical fitness and daily living.

Two possible reasons have been attributed to the decrease in motivation during hospitalization. First is the influence of high expectations during the early phase of hospitalization. Patients admitted to KRWs may often have higher expectations for improvement. A previous qualitative study of patients with various diseases admitted to a rehabilitation ward reported that 79% of patient goals set by occupational therapists were mismatched with those assumed by the patients themselves (33). During the early phase of hospitalization, when information from medical staff is scarce and rehabilitation has not been fully experienced, patients may tend to have high expectations of the goals they hope to achieve. In this study, the first motivational assessment was conducted relatively early in the admission process (median nine days after admission), when the expectations for the future were considered high, resulting in high MORE scores. The rehabilitation might not meet the patients’ high expectations of improvement. One of the factors that may cause motivation to change is the discrepancy between the predicted and the actual rewards when expectations and reality do not align (34). Motivation increases if the reward obtained is greater than the initially expected reward. Conversely, motivation decreases if the reward obtained is smaller than the initially expected reward. In this study, it is possible that some participants who initially expected to benefit from intensive training in the rehabilitation ward may have felt that their actual training did not meet their expectations for improvement. As a result, they experienced negative prediction errors, leading to a shift from a growth mindset (35), where they believed their abilities would improve through rehabilitation, to a fixed mindset (35), questioning the effectiveness of rehabilitation. This shift in mindset may have contributed to a decline in motivation. The results indicate that it is essential to establish effective communication between the medical staff and patients from the initial stages of hospitalization to keep patients motivated. Second, the motivation of hospitalized stroke patients is influenced by personal and social relationship factors (3). If the hospitalization period is prolonged, factors such as other patients being discharged from the same room earlier than they are and personal relationship stress among patients may decrease the motivation for rehabilitation.

The results of the present study showed that motivation tended to decrease at two and three months of hospitalization; however, the median converted motivation in stroke patients for rehabilitation scale scores of participants was 81.9 (74.8–95.1) at two and 81.4 (71.3–93.1) at three months after admission. Many participants remained highly motivated. Early subacute stroke patients, who experienced potential improvements of physical functions through continuous training and intensive rehabilitation in KRWs, were more likely to maintain high motivation because of the highly rewarding environment.

The results of this study were based on stroke patients with relatively high physical function who were admitted to a convalescent rehabilitation hospital. Our study has several limitations that may affect the generalizability of our results to all stroke patients. First, it involved stroke patients who met admission criteria to a single facility (KRWs) in Japan. Since KRWs give patients intensive rehabilitation, potentially highly motivated patients may have been admitted. Second, the participants were patients with a relatively high ability to perform activities of daily living as assessed by the FIM. The average FIM score of patients with cerebrovascular disease admitted to rehabilitation hospitals in Japan at the time of admission was 67.7 (21), and those included in this study tended to have scores higher than that. Therefore, we could not examine how motivation for rehabilitation affects the outcomes in stroke patients with relatively low independence in activities of daily living. Third, study participation was limited to patients with sufficient cognitive functioning to respond to the MORE scale. The study was not validated in stroke patients with aphasia or cognitive disability that could interfere with the measurement of the MORE scale score. Future studies should be multicenter trials, including stroke patients with low ability to perform activities of daily living and relatively low motivation, as well as outpatients. In addition, although the FIM was used as the outcome in this study, additional validation is needed with indices of physical fitness, activity level, and physical functions such as the stroke impairment assessment set, which are not directly reflected in FIM. Furthermore, while motivation was assessed once a month using the MORE scale, further investigation using methods such as ecological momentary assessment (36, 37) is necessary to enhance the resolution of motivation states.

### 4.1. Conclusion

The motivation for the rehabilitation of stroke patients with relatively high levels of physical activity admitted to the convalescent rehabilitation ward gradually decreased when they were hospitalized for a long time (more than two months). However, majority of patients maintained their motivation level for training during hospitalization. Motivation, as measured by the MORE scale at each assessment point, was not significantly associated with the FIM. Therefore, future studies must examine how physical activity is affected by motivation, especially in those with low motivational status, to provide a more comprehensive clarification regarding the influence of motivation on rehabilitation outcomes.

## Data availability statement

The original contributions presented in the study are included in the article/Supplementary material, further inquiries can be directed to the corresponding author.

## Ethics statement

The protocol of this study was reviewed and approved by Ethics Committee of the Tokyo Bay Rehabilitation Hospital (approval number: 144; date of approval: October 13, 2016) and Ethics Committee of Waseda University (approval number: 2019-059; date of approval: June 23, 2019). The participants provided their written informed consent to participate in this study.

## Author contributions

TY, YO, SK, KU, and MK provided substantial contributions to the study conception, design, acquisition, analysis, and interpretation. JY and RO contributed to the data acquisition. TY, YO, SK, KU, MK, JY, and RO contributed to the manuscript draft. TY, YO, and RO critically revised the manuscript. All authors contributed to the article and approved the submitted version.

## Funding

This study was supported by JSPS KAKENHI Grant-in-Aid for Young Scientists (Start-up) [grant no. 20K23271], Grant-in-Aid for Early-Career Scientists [grant no. 22K17579], and Grants-in-Aid for Scientific Research (KAKENHI) on Innovative Areas (Hyper-Adaptability) [grant no. 20H05482] from the Ministry of Education, Culture, Sports, Science, and Technology (MEXT), Japan. The funding sources had no role in the study design; in the acquisition, analysis, and interpretation of data; and in drafting the manuscript.

## Conflict of interest

The authors declare that the research was conducted in the absence of any commercial or financial relationships that could be construed as a potential conflict of interest.

## Publisher’s note

All claims expressed in this article are solely those of the authors and do not necessarily represent those of their affiliated organizations, or those of the publisher, the editors and the reviewers. Any product that may be evaluated in this article, or claim that may be made by its manufacturer, is not guaranteed or endorsed by the publisher.

## Abbreviations

CI: confidence interval
COSMIN: consensus-based standards for the selection of health measurement instruments
FIM: functional independence measure
IQR: interquartile range
KRWs: Kaifukuki Rehabilitation Wards
MORE scale: motivation in stroke patients for rehabilitation scale
