# Supplementary material for: Influence of motivation on rehabilitation outcomes after subacute stroke in convalescent rehabilitation wards
Source: Front Neurol. 2023 Jul 14;14:1185813. doi: 10.3389/fneur.2023.1185813 (PMC10375291; doi:10.3389/fneur.2023.1185813)
Supplement: Supplementary file 2 [file Data_Sheet_1.PDF]

Motivation in stroke patients for rehabilitation scale (MORE scale)

		Strongly disagree	Disagree	Somewhat disagree	Neither agree nor disagree	Some what agree	Agree	Strongly agree
1	I want to participate in rehabilitation for my goals.	1	2	3	4	5	6	7
2	I do not want to be discharged until I achieve my recovery objectives.	1	2	3	4	5	6	7
3	I want to train in order to regain my role in my home and our society.	1	2	3	4	5	6	7
4	I am able to make efforts to achieve my goal.	1	2	3	4	5	6	7
5	I want to work hard to meet my therapists' expectations.	1	2	3	4	5	6	7
6	I want to use the abilities I regained from the rehabilitation process in my daily	1	2	3	4	5	6	7
7	I share my daily exercise target with my therapist on a daily basis.	1	2	3	4	5	6	7
8	Alternations of daily rehabilitation plans propel me to participate more.	1	2	3	4	5	6	7
9	I was encouraged by other patients' efforts.	1	2	3	4	5	6	7
10	I want to participate in rehabilitation for the sake of my friends and family.	1	2	3	4	5	6	7
11	I feel my body functions (such as body movement) improve on a daily basis.	1	2	3	4	5	6	7
12	I would like to keep practicing so that I can regain my ability to perform lost/unexecuted daily activities.	1	2	3	4	5	6	7
13	I want to try several different exercises/practices.	1	2	3	4	5	6	7
14	I want to undergo the rehabilitation, even if I feel some pain and/or numbness	1	2	3	4	5	6	7
15	I want to train by myself in addition to usual supervised training.	1	2	3	4	5	6	7
16	I think I must actively participate in rehabilitation.	1	2	3	4	5	6	7
17	I think rehabilitation is essential for recovering from diseases and disabilities.	1	2	3	4	5	6	7
